# Sulbactam-enhanced cytotoxicity of doxorubicin in breast cancer cells

**DOI:** 10.1186/s12935-018-0625-9

**Published:** 2018-09-04

**Authors:** Shao-hsuan Wen, Shey-chiang Su, Bo-huang Liou, Cheng-hao Lin, Kuan-rong Lee

**Affiliations:** 1 Department of Molecular Medicine and Institute of Life Science, National Tsing Hua University, No. 101, Section 2, Kuang-Fu Road, Hsinchu, 30013 Taiwan, ROC; 2 Department of Internal Medicine, Puli Christian Hospital, No. 1, Tieshan Road, Puli Township, Nantou, 54546 Taiwan, ROC; 3 Department of Internal Medicine, Hsinchu Mackay Memorial Hospital, No.690, Section 2, Guangfu Road, East District, Hsinchu, 300 Taiwan, ROC

**Keywords:** Sulbactam, Breast cancer, ABC transporters, Doxorubicin, Proteomics, Inhibitors

## Abstract

**Background:**

Multidrug resistance (MDR) is a major obstacle in breast cancer treatment. The predominant mechanism underlying MDR is an increase in the activity of adenosine triphosphate (ATP)-dependent drug efflux transporters. Sulbactam, a β-lactamase inhibitor, is generally combined with β-lactam antibiotics for treating bacterial infections. However, sulbactam alone can be used to treat *Acinetobacter baumannii* infections because it inhibits the expression of ATP-binding cassette (ABC) transporter proteins. This is the first study to report the effects of sulbactam on mammalian cells.

**Methods:**

We used the breast cancer cell lines as a model system to determine whether sulbactam affects cancer cells. The cell viabilities in the present of doxorubicin with or without sulbactam were measured by MTT assay. Protein identities and the changes in protein expression levels in the cells after sulbactam and doxorubicin treatment were determined using LC–MS/MS. Real-time reverse transcription polymerase chain reaction (real-time RT-PCR) was used to analyze the change in mRNA expression levels of ABC transporters after treatment of doxorubicin with or without sulbactam. The efflux of doxorubicin was measures by the doxorubicin efflux assay.

**Results:**

MTT assay revealed that sulbactam enhanced the cytotoxicity of doxorubicin in breast cancer cells. The results of proteomics showed that ABC transporter proteins and proteins associated with the process of transcription and initiation of translation were reduced. The mRNA expression levels of ABC transporters were also decreased when treated with doxorubicin and sulbactam. The doxorubicin efflux assay showed that sulbactam treatment inhibited doxorubicin efflux.

**Conclusions:**

The combination of sulbactam and doxorubicin enhances the cytotoxicity of doxorubicin in the breast cancer cells by inhibiting the expression of ABC transporter proteins and proteins associated with the process of transcription and initiation of translation, and blocking the efflux of doxorubicin. Co-treatment of doxorubicin and sulbactam can be used in breast cancer treatment to decrease the prescribed dose of doxorubicin to avoid the adverse effects of doxorubicin.

## Background

Breast cancer, the most common cancer in women, annually affects 1.8 million women worldwide [1]. Approximately 12% of women in the United States are estimated to receive diagnoses of breast cancer in their lifetime [2]. Breast cancer is classified into three subtypes according to the expression of receptors: hormone (estrogen and progesterone)-receptor-positive breast cancer, human epidermal growth factor receptor 2 (HER2)-positive breast cancer, and triple-negative breast cancer (TNBC; lacking hormone receptors as well as HER2) [3]. Patients with TNBC exhibit a high risk of early tumor recurrence and poor prognosis [4]. Chemotherapy is a principal treatment for breast cancer, but resistance to chemotherapy—occurring in at least a quarter of all cases—is a major problem in breast cancer management, causing treatment failure in more than 90% of patients with metastatic cancers [5–8]. The mechanisms underlying resistance in different breast cancer subtypes are diverse, complex, and unclear. Cancer cells may develop resistance to a specific class of cytotoxic drugs owing to changes in target proteins and in cellular biological activities affecting the efficacy of the drugs. The changes include increased repair of DNA damage and decreased apoptosis, membrane permeability, and drug metabolism. Furthermore, the uptake of water-soluble drugs decreases due to a decrease in the expression of transporter proteins responsible for drugs to enter the cells and the energy-dependent efflux of hydrophobic drugs increases, for instance, through increased expression of adenosine triphosphate (ATP)-binding cassette (ABC) transporter proteins [9–15].

Doxorubicin, an anthracycline antibiotic, has been considered one of the most effective agents in breast cancer treatment since the 1970s [16]. Doxorubicin mainly intercalates between DNA bases and subsequently inhibits topoisomerase II activity, thus impairing DNA synthesis [17]. Doxorubicin also generates free radicals, which damage DNA and cell membranes [18]. Doxorubicin enters the cells through passive diffusion and accumulates intracellularly, particularly in the nuclear compartments [19]. However, doxorubicin is nonselective toward cancer cells; thus, it causes toxicity in the heart, brain, liver, and kidneys [19, 20]. The most prominent adverse event is life-threatening cardiotoxicity, which limits the prescribed dose of doxorubicin [20]. Doxorubicin resistance is another crucial cause of treatment failure [3]. The reported response rates to doxorubicin as a single agent for breast cancer treatment were 43% and 28% in patients who were exposed to doxorubicin for the first time and those who had been exposed to the drug for more than once, respectively. Thus, nearly 50% of the treated patients developed resistance to doxorubicin, making resistance the major cause of treatment failure [21]. The predominant mechanism underlying resistance to doxorubicin in breast cancer cells is the overexpression of a few ABC transporter proteins that increase doxorubicin efflux, thus decreasing intracellular drug concentrations [3, 9, 22]. Other mechanisms underlying doxorubicin resistance include alterations in cellular signaling pathways, leading to failure of apoptosis, and changes in gene expression, resulting in a chemoresistant phenotype [3, 19].

Increased expression of ABC transporter proteins has been correlated with poor clinical prognosis in patients with breast cancer of any subtype [23, 24]. The human genome has 49 members of the ABC transporter family, divided into seven subfamilies (ABCA–ABCG) based on their sequence similarities [25]. These membrane proteins actively pump various structurally and functionally diverse amphipathic anticancer drugs from inside the tumor cells to the outside, thereby decreasing intracellular drug concentrations and causing chemotherapeutic drug resistance [9, 10]. The primary members of the ABC transporter family leading to doxorubicin resistance in cancer cells are the ABCBs, the ABCCs [also known as multidrug resistance (MDR)-associated proteins], and ABCG2 (also known as breast cancer resistance protein, mitoxantrone resistance protein, or placenta-specific ABC transporter) [9, 26, 27]. Among the aforementioned ABC transporter proteins, ABCB1 [a P-glycoprotein, (p-gp)], ABCC1, and ABCG2 have been extensively characterized in breast cancers [23, 24, 28, 29]. Inhibitors of the ABC transporter proteins activity were used to overcome ABC transporter-mediated MDR for obstructing the expression of the transporter proteins or inhibiting their function. For example, a combination of doxorubicin and verapamil, a P-gp inhibitor, can reverse the resistance of breast cancer cells to doxorubicin [30]. However, verapamil can potentiate the cardiotoxicity of doxorubicin [31]. Over the past decades, numerous inhibitors of MDR-related ABC transporter proteins have been developed and identified. However, the development of most inhibitors has been discontinued because of their low binding affinity, toxicity, detrimental pharmacokinetic interactions, and low patient survival advantages [9, 32]. Furthermore, the expression patterns of ABC transporter proteins in breast cancer cells are heterogeneous; thus, the efficacy of inhibitors specific to some ABC transporter proteins is low [33].

Sulbactam, a β-lactamase inhibitor belonging to Ambler class A, is administered along with β-lactam antibiotics (e.g., ampicillin and penicillin) to prevent the hydrolysis of the antibiotics by bacterial β-lactamases. Sulbactam inhibits the activity of β-lactamases by irreversibly binding to their active sites. The β-lactam/β-lactamase inhibitor combination has been approved by the US Food and Drug Administration for treating dermatological, gynecological, and intraabdominal infections [34]. Although sulbactam has relatively low intrinsic biological activity, it has inherent activity against some bacterial species, including *Neisseria gonorrhoeae*, *Bacteroides fragilis*, and *Acinetobacter* spp. [35, 36]. Preliminary in vitro experiments have demonstrated that sulbactam kills bacteria by binding to the penicillin-binding proteins (PBPs) of *Acinetobacter* spp. and downregulating the expression of PBP1 and PBP3 [35, 37]. Furthermore, sulbactam reduces the expression of the ABC transporter proteins in *Acinetobacter baumannii* [38]. The ABC transporter superfamilies are highly conserved protein families, and their structural features and mechanisms of action have been conserved from prokaryotes to humans [39, 40]. Thus, we hypothesized that if sulbactam can reduce the expression of ABC transporter proteins in breast cancer cells, then it can reduce the efflux of doxorubicin from breast cancer cells and enhance its efficacy.

## Materials and methods

### Reagents

Doxorubicin hydrochloride was purchased from Sigma-Aldrich (St. Louis, MO, USA). Sulbactum sodium was obtained from TTY Biopharm (Taiwan). Verapamil was obtained from Orion Pharma (Espoo, Finland).

### Cell lines and cell culture

The breast carcinoma cell lines MDA-MB-231, MDA-MB-435, MDA-MB-453, and MDA-MB-468 were maintained in Dulbecco’s modified Eagle’s medium (DMEM) (Hyclone, Thermo Fisher Scientific Inc. Waltham, MA, USA) containing 10% fetal bovine serum (FBS; Gibco-BRL, Rockville, MD, USA) and 100 units/mL penicillin–streptomycin (Gibco-BRL). The breast carcinoma cell lines MCF-7, BT474, and T-47D were maintained in Roswell Park Memorial Institute (RPMI)-1640 medium (Hyclone) containing 10% FBS and 100 units/mL penicillin–streptomycin. The human breast epithelial cell line MCF-10A was maintained in DMEM/F12 medium containing 5% horse serum (Invitrogen, Carlsbad, CA, USA), 20 ng/mL epithelial growth factor (Peprotech, Rocky Hill, NJ, USA), 0.5 μg/mL hydrocortisone (Sigma-Aldrich), 10 μg/mL insulin (Sigma-Aldrich), and 100 units/mL penicillin–streptomycin. All cell lines were incubated at 37 °C and 5% CO_2_.

### MTT assay

The MTT (3-(4,5-dimethylthiazol-2-yl)-2,5-diphenyltetrazolium bromide) assay was used to access cytotoxicity. The cells were grown in 96-well plates at a density of 1.5 × 10^4^ cells/well. To determine the toxicities of sulbactam and doxorubicin, sulbactam and doxorubicin were added at various concentrations into the wells. At 48 h after treatment, the medium in the wells was replaced with 100 µL/well of medium containing 0.5 µg/µL MTT and incubated for 4 h. Subsequently, the medium was removed and 100 µL DMSO was added in each well to dissolve the formazan crystals. The absorbance of the samples was measured at 550 and 655 nm as the test and reference wavelengths, respectively, by using an iMark microplate reader (Bio-Rad, Hercules, CA, USA). To determine the effects of the combination of sulbactam and doxorubicin, various concentrations of doxorubicin were added to the medium containing 2 mM sulbactam in 96-well plates seeded with the breast cancer cells. The MTT assay was performed as described above. The cytotoxicity was expressed as relative viability (percentage of control). The percentage of cell survival in the negative control (without sulbactam and doxorubicin treatment) was considered 100. Relative viability = [(experimental absorbance − background absorbance)/(absorbance of untreated control − background absorbance)] × 100%. The half maximal inhibitory concentration (IC_50_) values of sulbactam, doxorubicin, and the combinations of sulbactam and doxorubicin were calculated using the survival curves by using the Bliss method. The degree of resistance was calculated by determining the ratio of the IC_50_ of the cells treated with sulbactam–doxorubicin combinations to that of the cells treated with doxorubicin alone.

### Real-time RT-PCR

Total RNA was extracted using TriZol (Invitrogen) and reverse transcribed (SuperScript III reverse transcriptase, Invitrogen and ExcelRT Reverse Transcriptase RP1000, SMOBIO, Taiwan). Real-time reverse transcription polymerase chain reaction (Real-time RT-PCR) was performed on ABI StepOnePlus™ Real-Time system using the SYBR Green PCR Master Mix (Applied Biosystems). The sequences of the PCR primers were listed in Table 1. The condition for PCR was 95 °C for 10 min, followed by 40 rounds of 95 °C for 15 s and 60 °C for 1 min. The data were analyzed by StepOne Software v2.2.2.

**Table 1:** List of primers of ABC transporters used for real-time RT-PCR

Gene	RefSeq	Forward oligo sequence	Reverse oligo sequence
ABCB1	NM_000927	AGCTCGTGCCCTTGTTAGACA	GTCCAGGGCTTCTTGGACAA
ABCB5	NM_178559	CACAAAAGGCCATTCAGGCT	GCTGAGGAATCCACCCAATCT
ABCB8	NM_007188	CATCGCCTTCAACTGCATGG	GACCTTTGCACTGTCTGGGA
ABCB10	NM_012089	TGCGGTTGGATTTCTCACGA	CACACAGAAACACGGCACTG
ABCC1	NM_004996	CGCTCTGGGACTGGAATGT	AGGTAAAAACAAGGCACCCA
ABCC2	NM_000392	TGCACAAGCAACTGCTGAAC	CCTCTGGCCTATGCTCAGGTT
ABCC3	NM_020038	ACCCAGTTTGATACCTGCACTGT	GGACCCTGGTGTAGTCCATGA
ABCC4	NM_005845	TTGGACACGGTAACTGTTGCA	GGAATGTCGGTTAGAGGTTTGG
ABCC5	NM_005688	ATTTGGACCCCTTCAACCAGTAC	GGTAGCTGAGCAATACATTCTTTCAT
ABCC10	NM_033450	CCTGTTGTTGGTGCTCTTCC	GGCCCTGTCCTTATGTAGGC
ABCG2	NM_004827	TATAGCTCAGATCATTGTCACAGTC	GTTGGTCGTCAGGAAGAAGAG
GAPDH	NM_002046	CCACCCATGGCAAATTCC	TCGCTCCTGGAAGATGGTG

### Efflux assay of doxorubicin

The MDA-MB-453 and MDA-MB-468 cells were seeded on coverslips in 12-well plates at a concentration of 1 × 10^5^ cells/well and grown for 16 h. On the following day, the cells were washed with phosphate buffered saline (PBS) and incubated with 2 mM sulbactam or 5 µM verapamil for 30 min before treating them with 2 µM doxorubicin for 2 h. The cells were subsequently incubated in a doxorubicin-free medium for 0, 8, 12, and 16 h. Images were obtained using a LSM 780 confocal microscope (Zeiss) and analyzed using ZEN 2012.

### Gel electrophoresis

The equivalence of the human cell lines was analyzed through 12.5% sodium dodecyl sulfate–polyacrylamide gel electrophoresis (SDS-PAGE). The gels were then stained using the VisPRO protein stain kit (Visual Protein Biotech, Taiwan) for 5 min. After staining, the gels were washed with Milli-Q water and stored at 4 °C until in-gel digestion.

### In-gel digestion

The gel lanes corresponding to the samples were cut into five slices, and each slice was subjected to in-gel digestion according to the method of Shevchenko [41]. Briefly, the slices were washed thrice with 50 mM ammonium bicarbonate (pH 7.9) and dehydrated using 50 mM AMBC + 50% acetonitrile (ACN). Subsequently, the cysteine bonds were reduced after treatment with 10 mM dithiothreitol for 1 h at 56 °C and alkylated using 50 mM 4-vinylpyridine for 45 min at room temperature in the dark. After two subsequent wash–dehydration cycles, the slices were dried for 10 min in a vacuum centrifuge (ThermoFisher, Breda, Netherlands) and incubated overnight with 6.25 ng/μL trypsin in 50 mM AMBC at 25 °C. The resulting peptides were extracted once in 100 μL of 1% formic acid and then two times in 100 μL of 50% ACN in 5% formic acid. The volume was reduced to 50 μL in a vacuum centrifuge before liquid chromatography (LC)–tandem mass spectrometry (MS/MS) analysis.

### LC–MS/MS

The peptides were separated using an Ultimate 3000 nano LC system (Dionex LC-Packings, Amsterdam, Netherlands) equipped with a 20 cm × 75 μm internal diameter (i.d.) fused-silica column custom packed with 3-μm 120-Å ReproSil Pur C18 aqua (Dr. Maisch, GMBH, Ammerbuch-Entringen, Germany). After injection, the peptides were delivered into the column at a flowrate of 30 μL/min and trapped on a 5 mm × 300 μm i.d. Pepmap C18 cartridge (Dionex LC-Packings), which were then eluted by 2% buffer B (80% ACN and 0.05% formic acid in Milli-Q water) and separated at 300 nL/min in a 10%–40% buffer B gradient within 60 min. The eluting peptides were ionized at 1.7 kV in a Nanomate Triversa Chip-based nanospray source by using a Triversa LC coupler (Advion, Ithaca, NJ, USA). Intact peptide mass spectra and fragmentation spectra were acquired on a LT QFT hybrid mass spectrometer (Thermo Fisher, Bremen, Germany). The intact masses were measured at a resolution of 50,000 in the ion cyclotron resonance (ICR) cell by using a target value of 1 × 10^6^ charges. Simultaneously, following an FT prescan, the five highest peptide signals (charge states 2+ and higher) were submitted for MS/MS in the linear ion trap (3-AMU isolation width, 30 ms activation, 35% normalized activation energy, 0.25 Q-value, and 5000-count threshold. Dynamic exclusion was applied with a repeat count of 1 and an exclusion time of 30 s.

## Results

### Sulbactam potentiates doxorubicin sensitivity in breast cancer cells

**Fig. 1:**
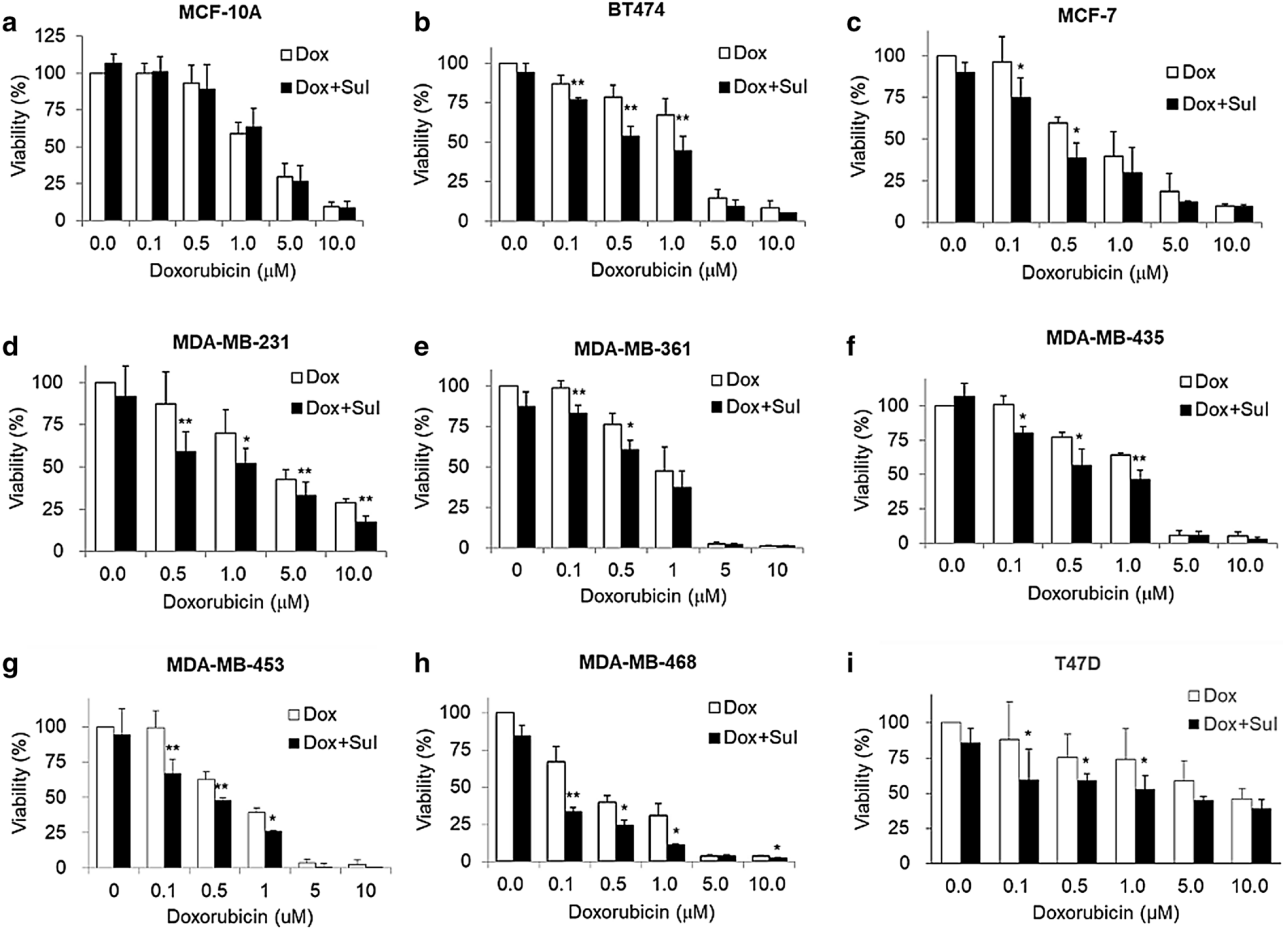
Treatment with a combination of sulbactam and doxorubicin reduced the viability of breast cancer cells. **a** MCF10A, **b** BT474, **c** MCF-7, **d** MDA-MB-231, **e** MDA-MB-361, **f** MDA-MB-435, **g** MDA-MB-453, **h** MDA-MB-468, **i** T47D. Data are expressed as the percentage of cell viability compared with the negative control in which the cell viability was assumed to be 100%. Reported values represent mean ± SD of at least three independent experiments. *p < 0.05 and **p < 0.01 versus only Dox-treated cells. *Sul* sulbactam, *Dox* doxorubicin, *ER* estrogen receptor, *PR* progesterone receptor, *HER2* human epidermal growth factor receptor 2, *MTT* 3-(4,5-dimethylthiazol-2-yl)-2,5-diphenyltetrazolium bromide, *SD* standard deviation

**Fig. 2:**
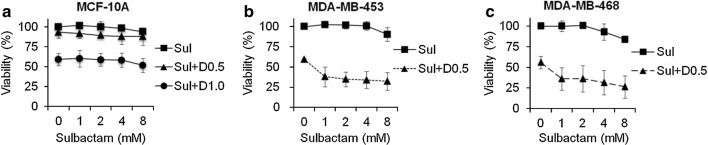
Sulbactam alone did not significantly affect cell viability of the breast cancer cell lines. **a** The MCF-10A cells treated with Sul (squares), Sul + D0.5 (triangles), and Sul + D1.0 (circles). The **b** MDA-MB-453 and **c** MDA-MB-468 cells treated with Sul (squares) and Sul + D0.5 (triangles). Data are expressed as the percentage of cell viability compared with negative control in which cell viability was assumed to be 100%. Reported values represent mean ± SD of at least three independent experiments. *Sul* sulbactam, *Dox* doxorubicin, *MTT* 3-(4,5-dimethylthiazol-2-yl)-2,5-diphenyltetrazolium bromide, *SD* standard deviation

To determine whether sulbactam enhances the cytotoxicity of doxorubicin, MCF-10A (normal), BT474 (ER/PR+, Her2+), MCF-7 (ER/PR+, Her2−), MDA-MB-231 (triple negative), MDA-MB-361 (ER/PR+, Her2+), MDA-MB-435 (ER/PR−, Her2+), MDA-MB-453 (triple negative), MDA-MB-468 (triple negative), and T47D (ER/PR+, Her2−) cell lines were treated for 48 h with 0, 0.1, 0.5, 1, 5, and 10 μM doxorubicin in the presence or absence of 2 mM sulbactam for 48 h. Cell viabilities were measured through the MTT assay. Doxorubicin exerted cytotoxic effects in a dose-dependent manner against all the cell lines (Fig. 1). When the cells were treated with doxorubicin alone, the viability of the MDA-MB-468 cells was < 50% at 0.5 µM doxorubicin, the viabilities of the MCF-7, MDA-MB-361, and MDA-MB-453 cells were < 50% at 1 μM doxorubicin, the viabilities of the BT474, MDA-MB-231, and MDA-MB-435 cells were < 50% at 5 μM doxorubicin, and the viability of T47D cells was < 50% until the concentration of doxorubicin reached 10 μM. Among these breast cancer cell lines, the T47D cell line exhibited low sensitivity to doxorubicin, with a IC50 value of 8.53 µM (Fig. 1i). By contrast, the MDA-MB-453 and MDA-MB-468 cells were more sensitive to doxorubicin than the T47D cells; they had lower IC_50_ values (0.69 and 0.27 μM, respectively) than the T47D cells and had the lowest viabilities at 5 and 10 μM doxorubicin (Fig. 1g, h). Next, we analyzed whether sulbactam enhanced the cytotoxicity of doxorubicin in the breast cancer cells. When the cells were treated with a combination of sulbactam and doxorubicin, the viabilities of the eight breast cancer cell lines significantly decreased (Fig. 1b–i). The IC_50_ values of doxorubicin in all the cell lines in the presence and absence of sulbactam are summarized in Table 2. The IC_50_ values of doxorubicin decreased from 1.14 to 0.54 μM in the BT474 cells, from 0.69 to 0.37 μM in the MCF-7 cells, from 3.16 to 1.25 μM in the MDA-MB-231 cells, from 0.89 to 0.46 μM in the MDA-MB-361 cells, from 1.22 to 0.51 μM in the MDA-MB-435 cells, from 0.69 to 0.27 μM in the MDA-MB-453 cells, from 0.27 to 0.05 μM in the MDA-MB-468 cells, and from 8.53 to 3.83 μM in the T47D cells in the presence of sulbactam. The IC_50_ of doxorubicin in breast cancer cells treated with a combination of sulbactam and doxorubicin was less than half of the IC_50_ of doxorubicin in the breast cancer cells treated with doxorubicin alone excluding the resistance of the MCF-7 and MDA-MB-361 cells, showed 1.85- and 1.96-fold decreases, respectively. By contrast, the MCF-10A cells (breast epithelial cells), did not exhibit evident differences in cell viability in the absence and presence of sulbactam; the IC_50_ values were 2.51 and 2.50, respectively (Fig. 1a). Among all the breast cancer cell lines, sulbactam considerably increased doxorubicin sensitivity in the MDA-MB-453 and MDA-MB-468 cells, by reducing the IC_50_ of doxorubicin by 2.6- and 5.0-fold, respectively, Subsequently, the cytotoxicity of sulbactam alone was analyzed in the MCF-10A, MDA-MB-453, and MDA-MB-468 cells. The cells were treated with 0, 1, 2, 4, and 8 mM sulbactam. Sulbactam did not exhibit an evident cytotoxic effect on any of the three cell lines at concentrations of up to 8 mM (Fig. 2). However, when combined with 0.5 μM doxorubicin, sulbactam potentiated the cytotoxicity of doxorubicin without evident dose dependence in the MDA-MB-453 and MDA-MB-468 cells. Thus, sulbactam has low cytotoxicity and can enhance the sensitivity of breast cancer cells toward doxorubicin.

**Table 2:** IC_50_ and resistance fold of breast cell lines in the present of sulbactam and doxorubicin

Cell line	IC_50_ of Doxorubicin (Dox, μM)		Resistance fold
	Dox	Dox + Sul	Dox + Sul/Dox
MCF10A	2.51	2.50	1.00
BT474	1.14	0.54	0.47
MCF-7	0.69	0.37	0.54
MDA-MB-231	3.16	1.25	0.40
MDA-MB-361	0.89	0.46	0.51
MDA-MB-435	1.22	0.51	0.42
MDA-MB-453	0.69	0.27	0.39
MDA-MB-468	0.27	0.05	0.20
T47D	8.53	3.83	0.45

IC_50_ was calculated from the results of Fig. 1 using CompuSyn. Resistance fold was determined by dividing the IC50 values of cells treated with doxorubicin and 2 mM sulbactam (Dox + Sul) by the IC50 of cells treated with doxorubicin (Dox)

### Proteomic profiling of total proteins from MDA-MB-468 cells treated with and without sulbactam in presence of doxorubicin

**Fig. 3:**
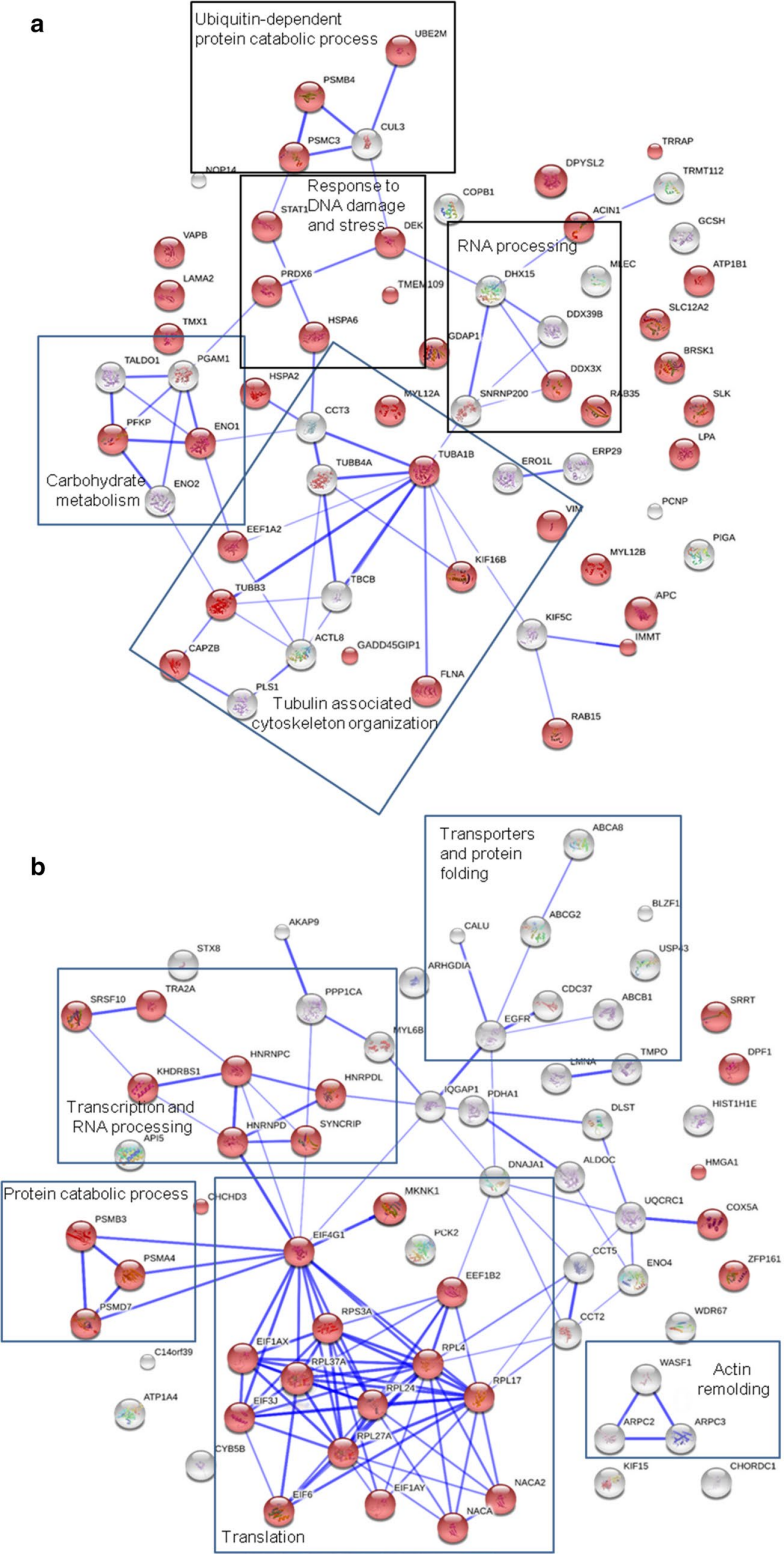
Differentially expressed proteins in the MDA-MB-468 cells in the presence of sulbactam and doxorubicin. Proteins are represented as nodes. **a** Upregulated proteins in the Dox/Sul-treated MDA-MB-468 cells. Red nodes indicate proteins that are related to the response to stimulus. **b** Downregulated proteins in the Dox/Sul-treated MDA-MB-468 cells. Red nodes indicate the proteins that are related to gene expression. *Sul* sulbactam, *Dox* doxorubicin

The MDA-MB-468 cells were treated with or without 2 mM sulbactam in the presence of 0.1 μM doxorubicin for 24 h. The total cell lysates were harvested for LC–MS/MS analysis. In total, 2937 proteins were identified using Sequest, which were validated using Scaffold. The expression of 66 and 70 proteins were significantly upregulated and downregulated, respectively, in the MDA-MB-468 cells treated with a combination of sulbactam and doxorubicin (based on p value < 0.05 and fold change > 2; Tables 3 and 4). The UniProt database was used to classify the identified proteins according to their biological processes. The upregulated proteins were classified as RNA processing, response to DNA damage, response to stress, cytoskeleton organization, protein folding, ubiquitin-dependent protein catabolic process, vesicle-mediated transport, carbohydrate metabolism, amino acid metabolism, and positive regulation of apoptosis proteins (Table 3). The downregulated proteins were classified as translation, regulation of transcription, RNA processing, ABC transporter, cytoskeleton organization, protein folding, protein catabolic process, carbohydrate metabolism, mitochondrial metabolic process, negative regulation of apoptosis, and signal transduction proteins (Table 4). The connections among the proteins and GO biological processes of the proteins were tested through STRING network analysis. The proteins are represented as nodes. The thickness of the edges indicates the strength of correlations between the proteins according to neighborhood, gene fusion, co-occurrence, co-expression, previous experiments, databases, and text-mining information at confidence scores higher than 0.5. As shown in Fig. 3a, 38 of the 60 proteins which were upregulated in the MDA-MB-468 cells treated with a combination of sulbactam and doxorubicin were associated with response to stimuli. Functional clusters included proteins involved in carbohydrate metabolism, tubulin-associated cytoskeleton organization, and ubiquitin-dependent protein catabolic process. As shown in Fig. 3b, 31 of 68 proteins which were downregulated in the MDA-MB-468 cells treated with a combination of sulbactam and doxorubicin were associated with gene expression. The functional clusters of these downregulated proteins were associated with actin remodeling, mitochondrial metabolic process, protein catabolic process, transcription and RNA process, and translation.

**Table 3:** List of upregulated proteins in the Dox- and Sul-treated MDA-MB-468 cells

Protein name	Abbreviation	UniProt ID	Mass (Da)	pI	Spectrum count		Dox + Sul/Dox	p value	Biological process
					Dox	Dox + Sul	Fold^a^		
Putative pre-mRNA-splicing factor ATP-dependent RNA helicase DHX15	DHX15	O43143	90,932.8	7.1	0.00	0.82	100.00	2.57E−07	RNA processing
U5 small nuclear ribonucleoprotein 200 kDa helicase	SNRNP200	O75643	244,507.6	5.7	0.00	1.10	100.00	2.41E−02	RNA processing
Spliceosome RNA helicase DDX39B	DDX39B	Q5STU3	48,826.1	7.2	0.00	1.85	100.00	2.52E−05	RNA processing
ATP-dependent RNA helicase DDX3X	DDX3X	O00571	73,112.2	6.7	0.00	1.39	100.00	7.43E−04	RNA processing
Nucleolar protein 14	NOP14	P78316	97,668.7	9.1	0.00	0.96	100.00	1.50E−03	RNA processing
Growth arrest and DNA damage-inducible proteins-interacting protein 1	GADD45GIP1	Q8TAE8	25,383.9	9.5	0.00	1.09	100.00	2.70E−02	Response to DNA damage
26S protease regulatory subunit 6A	PSMC3	P17980	49,203.5	5.1	0.27	1.11	4.06	4.64E−02	Response to DNA damage
Proteasome subunit beta type-4	PSMB4	P28070	29,204.3	9.1	0.00	0.96	100.00	1.08E−03	Response to DNA damage
Transformation/transcription domain-associated protein	TRRAP	Q9Y4A5	437,601.8	9.1	0.00	0.55	100.00	3.412E−05	Response to DNA damage
Protein DEK	DEK	P35659	42,674.4	9.3	0.00	1.53	100.00	6.37E−03	Response to DNA damage
Serine/threonine-protein kinase BRSK1	BRSK1	Q8TDC3	85,087.0	9.5	0.00	0.55	100.00	2.57E−07	Response to DNA damage
Adenomatous polyposis coli protein	APC	E7EMH9	32,790.8	5.4	0.00	0.55	100.00	3.41E−05	Response to DNA damage
Dihydropyrimidinase-related protein 2	DPYSL2	Q16555	62,293.6	5.9	0.00	0.98	100.00	1.32E−02	Response to stress
Sodium/potassium-transporting ATPase subunit beta-1	ATP1B1	P05026	35,061.3	9.1	0.00	1.11	100.00	7.81E−03	Response to stress
ERO1-like protein alpha	ERO1L	Q96HE7	51,991.8	5.4	0.00	0.56	100.00	1.12E−04	Response to stress
STE20-like serine/threonine-protein kinase	SLK	Q9H2G2	142,695.4	3.7	0.00	0.70	100.00	2.30E−02	Response to stress
Heat shock-related 70 kDa protein 2	HSPA2	P54652	70,021.0	5.6	0.00	2.69	100.00	9.41E−04	Response to stress
Putative heat shock 70 kDa protein 7	HSPA6	P48741	40,244.4	7.7	0.00	3.08	100.00	5.50E−03	Response to stress
Lipoprotein, Lp(A)	LPA	Q1HP67	226,516.1	7.2	0.00	0.55	100.00	2.57E−07	Response to stress
Apolipoprotein(a)	LPA	P08519	501,319.8	7.2	0.00	0.55	100.00	2.57E−07	Response to stress
Peroxiredoxin-6	PRDX6	P30041	24,903.8	6.0	0.27	1.68	6.15	1.42E−02	Response to stress
Solute carrier family 12 member 2	SLC12A2	P55011	131,447.1	6.0	0.37	2.21	6.03	4.53E−03	Response to stress
Thioredoxin-related transmembrane protein 1	TMX1	Q9H3N1	31,791.3	3.7	0.00	0.83	100.00	2.70E−04	Response to stress
Transmembrane protein 109	TMEM109	Q9BVC6	26,210.1	11.2	0.00	0.56	100.00	2.70E−04	Response to stress
MICOS complex subunit MIC60	IMMT	Q16891	80,026.5	5.7	1.12	2.74	2.45	1.34E−04	Response to stress
Signal transducer and activator of transcription	STAT1	J3KPM9	83,360.6	7.2	0.00	0.83	100.00	2.70E−04	Response to stress
cDNA FLJ78587	TUBA1B	A8JZY9	50,135.7	5.4	4.80	14.97	3.12	5.22E−03	Cytoskeleton organization
Myosin regulatory light chain 12A	MYL12A	P19105	19,794.1	4.7	1.23	5.81	4.71	3.07E−02	Cytoskeleton organization
Myosin regulatory light chain 12B	MYL12B	O14950	19,779.2	4.7	1.23	5.81	4.71	3.07E−02	Cytoskeleton organization
Actin-like protein 8	ACTL8	Q9H568	41,360.4	7.2	0.27	1.11	4.06	4.64E−02	Cytoskeleton organization
Plastin-1	PLS1	Q14651	70,253.6	5.4	0.00	0.97	100.00	7.05E−03	Cytoskeleton organization
F-actin-capping protein subunit beta	CAPZB	P47756	31,219.3	5.4	0.00	2.46	100.00	4.59E−02	Cytoskeleton organization
Vimentin	VIM	B0YJC5	26,858.9	3.7	0.00	0.69	100.00	2.03E−02	Cytoskeleton organization
Filamin A	FLNA	Q60FE5	278,226.9	7.2	2.51	7.53	3.01	2.05E−02	Cytoskeleton organization
Tubulin-folding cofactor B	TBCB	Q99426	27,325.5	8.7	0.00	0.70	100.00	2.30E−02	Cytoskeleton organization
Tubulin beta-3 chain	TUBB3	Q13509	50,432.7	4.8	1.41	6.70	4.75	3.23E−02	Cytoskeleton organization
Tubulin beta-4A chain	TUBB4A	P04350	49,585.8	4.8	0.00	1.94	100.00	2.70E−04	Cytoskeleton organization
Kinesin heavy chain isoform 5C	KIF5C	O60282	109,494.8	5.9	0.00	1.10	100.00	3.85E−02	Cytoskeleton organization
Septin-9	SEPTIN9	Q9UHD8	65,401.6	9.5	0.00	1.40	100.00	1.64E−02	Cytoskeleton organization
Laminin subunit alpha-2	LAMA2	A0A087WYF1	343,419.0	7.2	0.28	1.26	4.46	4.32E−02	Cytoskeleton organization
Malectin	MLEC	Q14165	32,233.9	7.2	0.00	0.70	100.00	2.15E−02	Protein folding
T-complex protein 1 subunit gamma	CCT3	Q2TU64	60,579.1	7.2	0.00	3.62	100.00	1.49E−02	Protein folding
Vesicle-associated membrane protein-associated protein B/C	VAPB	E5RK64	7801.0	9.5	0.00	1.54	100.00	3.83E−02	Protein folding
PEST proteolytic signal-containing nuclear protein	PCNP	Q8WW12	18,924.9	6.9	0.28	1.91	6.95	2.46E−02	Ubiquitin-dependent protein catabolic process
NEDD8-conjugating enzyme Ubc12	UBE2 M	P61081	20,900.0	9.1	0.00	0.83	100.00	2.70E−04	Ubiquitin-dependent protein catabolic process
Cullin-3	CUL3	A0A087WTG3	39,147.2	9.5	0.00	1.94	100.00	2.70E−04	Ubiquitin-dependent protein catabolic process
Coatomer subunit beta	COPB1	P53618	107,142.6	7.2	0.00	1.10	100.00	3.02E−02	Vesicle-mediated transport
Endoplasmic reticulum resident protein 29	ERP29	F8VY02	18,115.9	9.1	0.00	0.55	100.00	3.41E−05	Vesicle-mediated transport
Kinesin-like protein KIF16B	KIF16B	Q96L93	152,011.7	7.2	0.00	1.12	100.00	3.33E−02	Vesicle-mediated transport
Phosphatidylinositol *N*-acetylglucosaminyltransferase subunit A	PIGA	P37287	54,126.7	9.1	0	0.55	100.00	2.574E−07	Vesicle-mediated transport
Ras-related protein Rab-35	RAB35	Q15286	23,025.3	9.1	0.00	0.98	100.00	8.01E−03	Vesicle-mediated transport
Ras-related protein Rab-15	RAB15	P59190	24,390.6	5.5	0.00	0.98	100.00	8.01E−03	Vesicle-mediated transport
Ras-related protein Rab-15 isoform AN2	RAB15	G5ELZ5	13,781.8	9.1	0.00	0.98	100.00	8.01E−03	Vesicle-mediated transport
Ras-related protein Rab-15 isoform AN3	RAB15	G5ELZ6	12,759.7	9.1	0.00	0.98	100.00	8.01E−03	Vesicle-mediated transport
Enolase	ENO1	F5H0C8	34,762.3	3.6	0.00	0.83	100.00	2.70E−04	Carbohydrate metabolism
Phosphoglycerate mutase	PGAM1	A4D2J6	28,219.6	9.5	0.00	1.40	100.00	2.15E−02	Carbohydrate metabolism
ATP-dependent 6-phosphofructokinase, platelet type	PFKP	B1APP8	22,939.3	9.1	0.00	0.56	100.00	1.12E−04	Carbohydrate metabolism
Gamma-enolase	ENO2	P09104	47,268.6	4.9	0.00	0.83	100.00	2.70E−04	Carbohydrate metabolism
Transaldolase	TALDO1	F2Z393	35,328.9	9.5	0.00	2.75	100.00	3.85E−02	Carbohydrate metabolism
Ganglioside-induced differentiation-associated protein 1	GDAP1	Q8TB36	41,345.8	9.1	0.00	0.56	100.00	2.70E−04	Amino acid metabolic process
Multifunctional methyltransferase subunit TRM112-like protein	TRMT112	F5GX77	11,972.0	7.8	0.00	0.56	100.00	1.12E−04	Amino acid metabolic process
GCSH protein	GCSH	Q6IAT2	19,025.8	3.7	0.00	0.96	100.00	8.84E−03	Amino acid metabolic process
Elongation factor 1-alpha 2	EEF1A2	Q05639	50,470.2	9.1	0.00	3.21	100.00	1.94E−02	Positive regulation of apoptotic process
Apoptotic chromatin condensation inducer in the nucleus	ACIN1	Q9UKV3	151,861.9	5.4	0.00	0.82	100.00	1.30E−02	Positive regulation of apoptotic process

*Sul* sulbactam, *Dox* doxorubicin

^a^The fold is from Dox + Sul/Dox, if the number of Dox is 0.00, the fold would be shown as 100.00

**Table 4:** List of downregulated proteins in the Dox- and Sul-treated MDA-MB-468 cells

Protein name	Abbreviation	UniProt ID	Mass (Da)	pI	Spectrum count		Dox + sul/Dox	p value	Biological process
					Dox	Dox + Sul	Fold^a^		
60S ribosomal protein L4	RPL4	P36578	47,566.1	11.1	4.53	1.96	− 2.31	2.25E−02	Translation
60S ribosomal protein L17	RPL17	J3QLC8	20,246.8	9.5	1.84	0.27	− 6.76	4.24E−02	Translation
60S ribosomal protein L24	RPL24	C9JXB8	14,368.8	11.3	0.56	0.00	− 100.00	1.30E−04	Translation
60S ribosomal protein L27a	RPL27A	P46776	16,430.2	11.0	3.20	1.47	− 2.17	1.42E−02	Translation
60S ribosomal protein L37a	RPL37A	P61513	10,275.3	9.5	1.39	0.00	− 100.00	4.08E−03	Translation
40S ribosomal protein S3a	RPS3A	D6RAT0	25,887.1	9.5	7.51	0.00	− 100.00	4.01E−03	Translation
Eukaryotic translation initiation factor 1A, Y-chromosomal	EIF1AY	O14602	16,442.4	4.6	0.65	0.00	− 100.00	1.43E−03	Translation
Eukaryotic translation initiation factor 1A, X-chromosomal	EIF1AX	P47813	16,460.4	4.6	0.65	0.00	− 100.00	1.43E−03	Translation
Eukaryotic translation initiation factor 4 gamma 1	EIF4G1	B2RU10	176,207.3	5.4	1.12	0.00	− 100.00	3.18E−02	Translation
Eukaryotic translation initiation factor 3 subunit J	EIF3 J	O75822	29,062.4	3.7	1.38	0.27	− 5.10	1.16E−02	Translation
Eukaryotic translation initiation factor 6	EIF6	P56537	26,599.2	3.7	0.70	0.00	− 100.00	2.17E−02	Translation
Nascent polypeptide-associated complex subunit alpha	NANA2	Q13765	23,383.9	4.5	1.57	0.00	− 100.00	1.47E−03	Translation
Nascent polypeptide-associated complex subunit alpha, muscle-specific form	NANA	F8VZJ2	15,016.0	4.9	1.57	0.00	− 100.00	1.47E−03	Translation
Eukaryotic translation elongation factor 1 beta 2	EEF1B2	A4D1M6	24,891.0	3.7	1.26	0.00	− 100.00	7.44E−03	Translation
Heterogeneous nuclear ribonucleoprotein D0	HNRNPD	Q14103	38,434.2	7.6	4.25	2.39	− 1.78	2.07E−02	Translation
MAP kinase-interacting serine/threonine-protein kinase 1	MKNK1	E9PMF1	12,586.3	9.5	0.84	0.00	− 100.00	8.80E−07	Translation
Heterogeneous nuclear ribonucleoproteins C1/C2	HNPNPC	G3V2Q1	33,570.9	5.0	3.50	0.81	− 4.30	2.30E−02	Translation
KH domain-containing, RNA-binding, signal transduction-associated protein 1	KHDRBS1	Q07666	48,227.3	8.7	0.97	0.00	− 100.00	1.21E−02	Regulation of transcription
High mobility group protein HMG-I/HMG-Y	HMGA1	P17096	11,544.8	10.3	1.53	0.70	− 2.19	2.87E−02	Regulation of transcription
cDNA FLJ54188, moderately similar to High mobility group protein HMG-I/HMG-Y	HMGA1	B4DWA0	34,301.4	10.4	1.53	0.70	− 2.19	2.87E−02	Regulation of transcription
Serrate RNA effector molecule homolog	SRRT	Q9BXP5	100,666.7	7.2	0.97	0.00	− 100.00	7.88E−03	Regulation of transcription
Protein SIX6OS1	C14orf39	Q8N1H7	68,166.0	5.4	0.56	0.00	− 100.00	1.30E−04	Regulation of transcription
Heterogeneous nuclear ribonucleoprotein D-like	HNRPDL	O14979	46,437.5	9.6	2.23	0.00	− 100.00	9.63E−03	Regulation of transcription
Zinc finger and BTB domain-containing protein 14	ZFP161	O43829	50,956.5	5.4	0.74	0.00	− 100.00	9.26E−03	Regulation of transcription
Golgin-45	BLZF1	Q9H2G9	44,910.4	9.1	0.70	0.00	− 100.00	1.68E−02	Regulation of transcription
Zinc finger protein neuro-d4	DPF1	E9PDV3	45,285.6	7.2	0.56	0.00	− 100.00	1.30E−04	Regulation of transcription
Histone cluster 1, H1e	HIST1H1E	Q4VB24	21,893.3	9.5	4.44	0.00	− 100.00	1.25E−02	Regulation of transcription
Serine/arginine-rich splicing factor 10	SRSF10	O75494	31,300.5	11.2	0.56	0.00	− 100.00	1.30E−04	RNA processing
Heterogeneous nuclear ribonucleoprotein Q	SYNCRIP	O60506	69,471.4	8.7	5.98	1.95	− 3.07	3.52E−02	RNA processing
Transformer-2 protein homolog alpha	TRA2A	Q13595	32,688.6	11.2	1.11	0.18	− 6.02	3.23E−02	RNA processing
Multidrug resistance protein 1	ABCB1	P08183	141,479.1	9.1	3.47	0.84	− 4.13	5.08E−04	Transporters
ATP-binding cassette sub-family G member 2	ABCG2	Q9UNQ0	72,314.0	8.9	1.66	0.36	− 4.56	1.05E−03	Transporters
ATP-binding cassette sub-family A member 8	ABCA8	O94911	179,245.9	9.1	0.56	0.00	− 100.00	8.80E−07	Transporters
Sodium/potassium-transporting ATPase subunit alpha-4	ATP1A4	E9PRA5	57,244.4	9.1	0.56	0.00	− 100.00	1.30E−04	Transporters
Syntaxin-8	STX8	Q9UNK0	26,906.8	3.7	0.56	0.00	− 100.00	8.80E−07	Transporters
Wiskott-Aldrich syndrome protein family member 1	WASF1	Q92558	61,652.4	5.4	0.56	0.00	− 100.00	1.30E−04	Cytoskeleton organization
Actin-related protein 2/3 complex subunit 2	ARPC2	O15144	34,333.1	9.1	1.23	0.00	− 100.00	5.68E−03	Cytoskeleton organization
Actin-related protein 2/3 complex subunit 3	ARPC3	O15145	20,415.5	8.8	0.97	0.00	− 100.00	7.88E−03	Cytoskeleton organization
Ras GTPase-activating-like protein IQGAP1	IQGAP1	P46940	189,120.8	6.1	1.25	0.00	− 100.00	4.32E−04	Cytoskeleton organization
Myosin light chain 6B	MYL6B	P14649	22764.1	6.3	2.23	0.00	− 100.00	8.80E−07	Cytoskeleton organization
TBC1 domain family member 31	WDR67	Q96DN5	124189.8	9.1	1.11	0.00	− 100.00	9.75E−04	Cytoskeleton organization
Prelamin-A/C	LMNA	Q5TCI8	55762.4	6.6	10.73	0.28	− 37.73	1.74E−02	Cytoskeleton organization
Lamin A/C	LMNA	W8QEH3	65116.9	9.1	11.69	0.00	− 100.00	8.86E−03	Cytoskeleton organization
Calumenin	CALU	O43852	34961.1	4.5	1.66	0.36	− 4.60	2.73E−02	Cytoskeleton organization
Lamina-associated polypeptide 2, isoforms beta/gamma	TMPO	P42167	50670.3	9.5	1.85	0.74	− 2.49	2.75E−02	Cytoskeleton organization
Kinesin-like protein	KIF15	A0A087X0P0	312105.2	5.5	2.19	0.00	− 100.00	6.76E−06	Cytoskeleton organization
DnaJ homolog subfamily A member 1	DNAJA1	P31689	44868.4	7.2	1.26	0.36	− 3.44	2.12E−02	Protein folding
T-complex protein 1 subunit epsilon	CCT5	P48643	59539.8	5.4	3.47	0.41	− 8.39	9.26E−03	Protein folding
T-complex protein 1 subunit beta	CCT2	P78371	57357.0	6.0	1.11	0.00	− 100.00	1.30E−04	Protein folding
Cysteine and histidine-rich domain-containing protein 1	CHORDC1	Q9UHD1	37489.9	7.2	0.55	0.00	− 100.00	6.76E−06	Protein folding
CDC37 protein	CDC37	Q6FG59	44453.5	3.7	1.78	0.41	− 4.38	4.29E−02	Protein folding
26S proteasome non-ATPase regulatory subunit 7	PSMD7	P51665	37025.4	6.3	0.69	0.00	− 100.00	1.95E−02	Protein catabolic process
Proteasome subunit beta type-3	PSMB3	P49720	22949.0	9.1	0.56	0.00	− 100.00	1.30E−04	Protein catabolic process
Proteasome subunit alpha type-4	PSMA4	P25789	29483.8	7.6	0.65	0.00	− 100.00	1.01E−03	Protein catabolic process
Ubiquitin carboxyl-terminal hydrolase 43	USP43	Q70EL4	122809.5	9.5	0.83	0.00	− 100.00	4.42E−02	Protein catabolic process
Enolase-like protein ENO4	ENO4	J3KNX1	68464.9	6.3	1.11	0.00	− 100.00	5.54E−03	Carbohydrate metabolism
PCK2 protein	PCK2	Q6IB91	70697.2	7.2	0.70	0.00	− 100.00	2.17E−02	Carbohydrate metabolism
Fructose-bisphosphate aldolase	ALDOC	A8MVZ9	36295.3	7.6	1.53	0.00	− 100.00	2.78E−03	Carbohydrate metabolism
Cytochrome c oxidase subunit 5A, mitochondrial	COX5A	H3BNX8	17234.9	7.2	1.24	0.00	− 100.00	4.42E−02	Mitochondrial metabolic process
Cytochrome b5 type B	CYB5B	J3KNF8	16694.6	6.3	0.82	0.00	− 100.00	4.69E−02	Mitochondrial metabolic process
Cytochrome b-c1 complex subunit 1, mitochondrial	UQCRC1	P31930	52646.0	7.2	0.56	0.00	− 100.00	1.30E−04	Mitochondrial metabolic process
MICOS complex subunit MIC19	CHCHD3	Q9NX63	26152.4	9.1	0.70	0.00	− 100.00	1.68E−02	Mitochondrial metabolic process
Phosphoenolpyruvate carboxykinase [GTP], mitochondrial	PCK2	Q16822	70730.2	7.2	0.70	0.00	− 100.00	2.17E−02	Mitochondrial metabolic process
Dihydrolipoyllysine-residue succinyltransferase component of 2-oxoglutarate dehydrogenase complex, mitochondrial	DLST	P36957	48755.5	9.5	0.56	0.00	− 100.00	1.30E−04	Mitochondrial metabolic process
Pyruvate dehydrogenase E1 component subunit alpha, somatic form, mitochondrial	PDHA1	P08559	43295.8	9.1	0.56	0.00	− 100.00	1.30E−04	Mitochondrial metabolic process
Apoptosis inhibitor 5	API5	Q9BZZ5	59004.7	9.1	1.21	0.27	− 4.44	4.79E−02	Negative regulation of apoptotic process
Epidermal growth factor receptor	EGFR	A9CB80	132022.7	6.2	6.03	0.54	− 11.12	5.65E−03	Signal transduction
A-kinase anchor protein 9	AKAP9	Q99996	453668.7	3.7	0.84	0.00	− 100.00	7.75E−05	Signal transduction
Rho GDP-dissociation inhibitor 1	ARHGDIA	J3KS60	9944.0	4.2	0.65	0.00	− 100.00	5.33E−03	Signal transduction
Serine/threonine-protein phosphatase PP1-alpha catalytic subunit	PPP1CA	P62136	37512.2	7.2	1.81	0.00	− 100.00	4.26E−03	Signal transduction

*Sul* sulbactam, *Dox* doxorubicin

^a^The fold is from Dox/Dox + Sul, and “−” means the expression of protein was decrease in Dox + Sul group. If the number of Dox + Sul is 0.00, the fold would be shown as − 100.00

### Sulbactam downregulates mRNA levels of ABC transporters in breast cancer cell lines

**Fig. 4:**
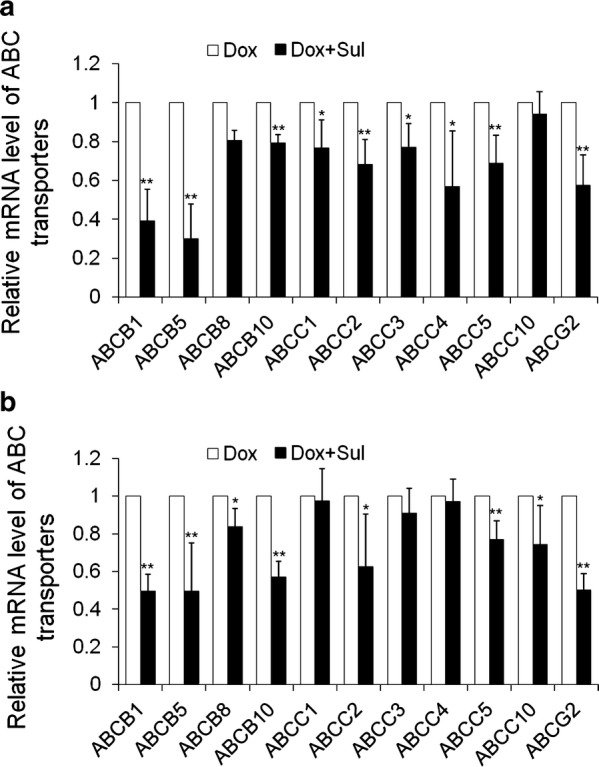
Co-treatment of sulbactam and doxorubicin downregulated mRNA expression levels of ABC transporters. **a** MDA-MB-453 and **b** MDA-MB-468. The relative mRNA expression levels are expressed as compared with Dox-treated cells where the mRNA expression levels were assumed to be 1. Reported values represent mean ± SD of at least three independent experiments, each performed in triplicate. *p < 0.05 and **p < 0.01 versus only Dox-treated cells. *Sul* sulbactam, *Dox* doxorubicin, *SD* standard deviation

Sulbactam significantly reduced ABC transporter protein expression in *A. baumannii* ATCC 19606. Breast cancer cells can actively remove doxorubicin from inside the cells by using ABC transporters to protect the cells from being killed by doxorubicin. LC–MS/MS results showed a reduction in the protein levels of ABCA8, ABCB1, and ABCG2; hence, we examined whether sulbactam can inhibit the mRNA expression of ABC transporters in the human breast cancer cells in the presence of doxorubicin. Two breast cancer cell lines, MDA-MB-453 and MDA-MB-468, were treated with 0.1 μM doxorubicin and 2 mM sulbactam for 24 h. The mRNA expression of the ABC transporters in these two cell lines were measured using real-time RT-PCR. In the presence of doxorubicin, sulbactam significantly reduced the mRNA expression of ABCB1, ABCB5, and ABCG2 by approximately 50% in the MDA-MB-453 and MDA-MB-468 cells (Fig. 4). Sulbactam also moderately reduced the mRNA expression of ABCB8, ABCB10, ABCC1, ABCC2, ABCC3, ABCC4, and ABCC5 in the MDA-MB-453 cells and those of ABCB8, ABCB10, ABCC2, ABCC5, and ABCC10 in the MDA-MB-468 cells by 20–30%. These results indicate that sulbactam downregulated the mRNA expression of several ABC transporters, particularly ABCB1, ABCB5, and ABCG2. These results also demonstrate that the combination of sulbactam and doxorubicin enhanced the sensitivity of the cells to doxorubicin by downregulating the expressions of ABC transporters related to the efflux of doxorubicin.

### Sulbactam prolongs doxorubicin retention in breast cancer cells

**Fig. 5:**
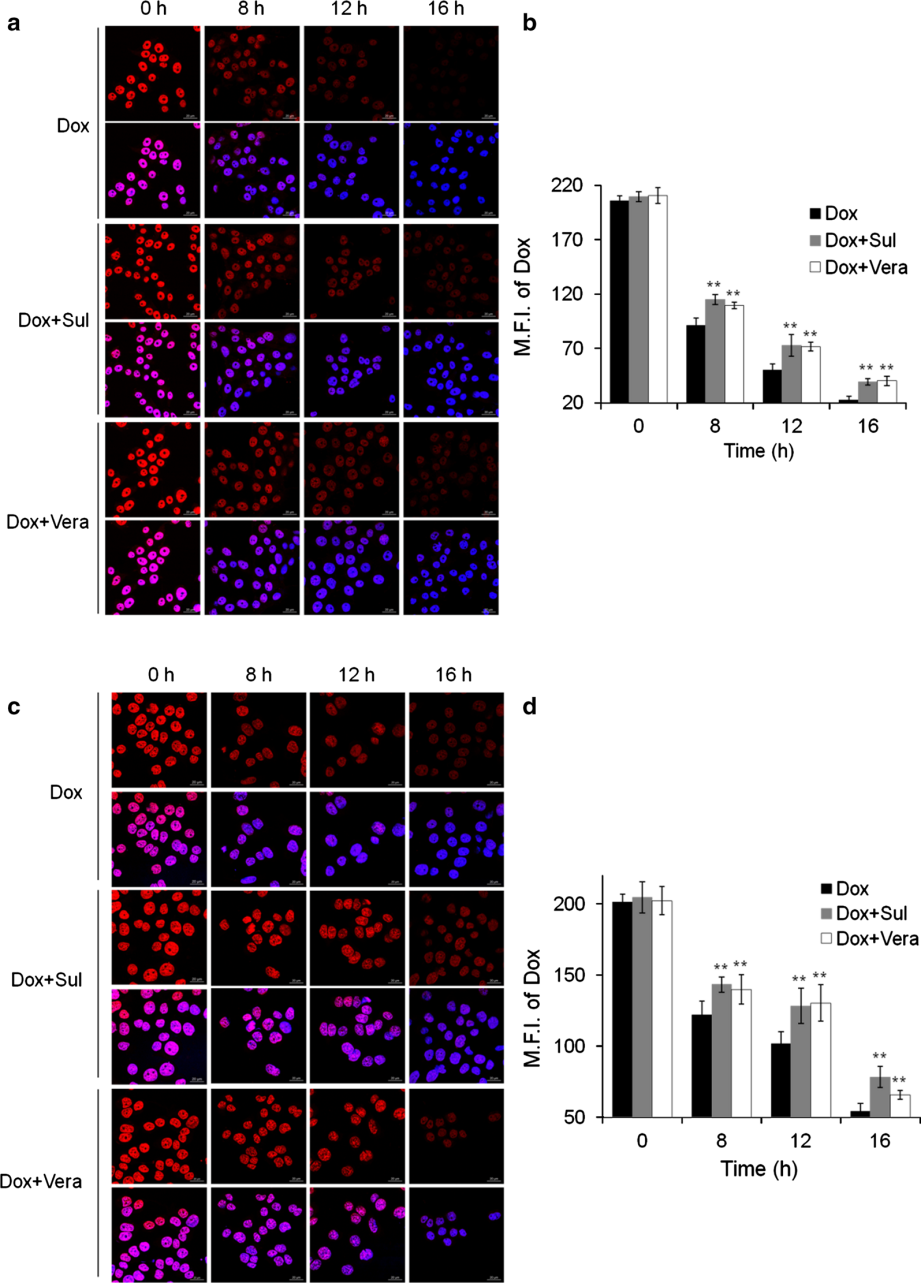
Prolonged doxorubicin retention in breast cancer cells in the presence of sulbactam. The distribution of Dox in the **a** MDA-MB-453 and **c** MDA-MB-468 cells was observed. Dox is shown in red and DAPI in blue, which counterstained the nuclei. Scale bars, 20 μm. **b**, **d** are quantifications of **a**, **c**, respectively. Reported values indicate the means of fluorescence intensity of Dox overlapping with DAPI and are represented as mean ± SD. **p < 0.01 versus only Dox-treated cells. *Sul* sulbactam, *Dox* doxorubicin, *SD* standard deviation, *Vera* verapamil, *DAPI* (4′,6-diamidino-2-phenylindole)

To investigate whether the sulbactam-induced reduction in the expression of ABC transporters inhibits the efflux of doxorubicin, the distribution of doxorubicin in breast cancer cells was observed using a confocal microscope. A time-course study was performed in the presence and absence of sulbactam. For comparison, the cells were also pretreated with verapamil, a well-known inhibitor of ABCB1 and ABCG2. The fluorescent signal corresponding to doxorubicin was primarily observed in nuclei of the cells, and the concentration of doxorubicin decreased time-dependently (Fig. 5). Pretreatment with sulbactam increased the doxorubicin concentration in the cell nuclei by 15, 45, and 74% in the MDA-MB-453 cells and 17, 26, and 44% in the MDA-MB-468 cells at 8, 12, and 16 h, respectively, compared with that in cells without sulbactam treatment. The intensities of doxorubicin were comparable between the sulbactam- and verapamil-treated MDA-MB-453 cells. Doxorubicin concentration was higher in the sulbactam-treated MDA-MB-468 cells than in the verapamil-treated cells. These results indicate that sulbactam inhibited the efflux of doxorubicin, thus prolonging doxorubicin retention in the breast cancer cells. The increase in intracellular doxorubicin levels resulted in an increase in its cytotoxicity in the breast cancer cells.

## Discussion

The coadministration of sulbactam and a β-lactam antibiotic, such as ampicillin, is an effective therapy against bacteria, such as *A. baumannii* [42]. Sulbactam alone has intrinsic bactericidal effects against multidrug-resistant *A. baumannii* because it inhibits the expression of the ABC transporters as well as that of 30S and 50S ribosomal subunit proteins [38]. However, the effects of sulbactam have not been explored in mammalian cells, thus far. Our study results suggest that sulbactam enhanced the cytotoxicity of doxorubicin in many of the tested breast cancer cell lines. Because of the high heterogeneity of breast cancer, we classified breast cancer cell lines as hormone-receptor-positive cancer, HER2-positive cancer, and TNBC; the cells were then treated with sulbactam and doxorubicin. All the cell lines responded to doxorubicin and sulbactam—a finding is evidently uncorrelated with the characteristic of these cell lines. Thus, a combination of doxorubicin and sulbactam exhibited the most significant cytotoxicity in the MDA-MB-453 and MDA-MB-468 cells. Dose-dependency tests showed that approximately 1–8 mM sulbactam was not cytotoxic to MDA-MB-453, MDA-MB-468, and MCF10A cells, which are typically used as normal breast cell lines; hence, in combination with doxorubicin, sulbactam exerted a synergistic effect on doxorubicin.

**Fig. 6:**
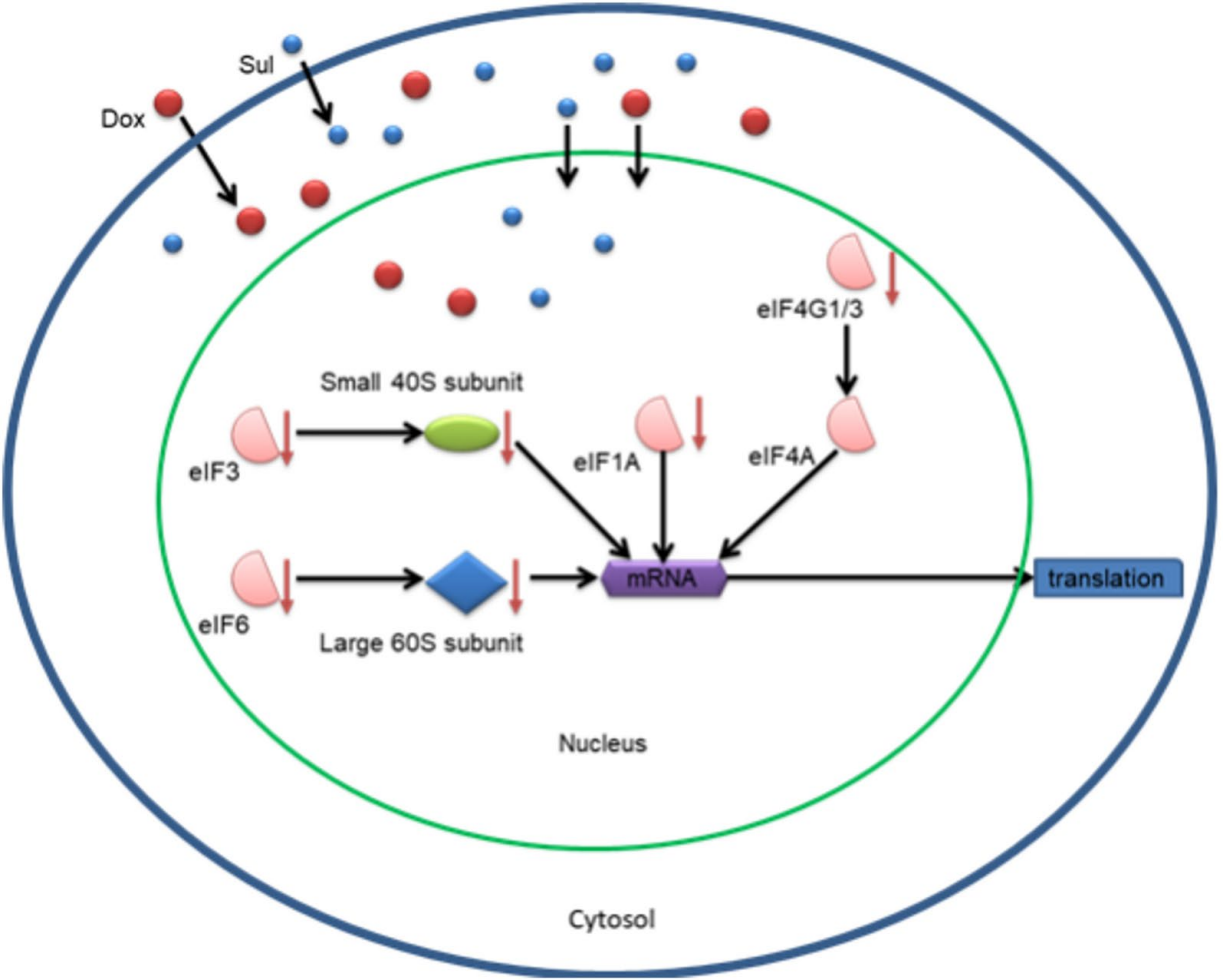
Co-treatment of sulbactam and doxorubicin blocked the initiation of translation in breast cancer cells. The illustration shows that treatment of the MDA-MB-468 cells with Sul (blue circles) and Dox (red circles) reduced the protein expression levels of eIF1A, eIF3, eIF4G1/3, eIF6, small 40S subunit, and large 60S subunit in the cells. Therefore, the transcription and initiation of translation pathways were blocked. *Sul* sulbactam, *Dox* doxorubicin

The results of LC–MS/MS indicated that most of the upregulated proteins (21/66) associated with stress and DNA damage response, such as heat shock-related 70-kDa protein 2 and adenomatous polyposis coli protein, may respond to the stress caused by sulbactam. When used as a drug, sulbactam also stimulates some metabolic pathways and cytoskeleton organizations, such as carbohydrate metabolism and tubulin-associated cytoskeleton organization. In the presence of doxorubicin and sulbactam evidently inhibited the initiation of RNA processing, transcription, and translation (Fig. 6). Doxorubicin interacts with DNA through intercalation between bases and macromolecular biosynthesis inhibition [19]. This inhibits the progression of topoisomerase II, which relaxes supercoils in DNA during transcription. Through intercalation, doxorubicin can also induce histone eviction from transcriptionally active chromatin [43]. Consequently, here, RNA processing and translation were downregulated in the doxorubicin-exposed cells. Sulbactam increased the doxorubicin retention time in the breast cancer cells. Therefore, in the presence of sulbactam, the effects of doxorubicin on transcription and translation were enhanced, and the 60S ribosomal proteins, namely L4, L17, L24, L37a, and 40S ribosomal protein 3A, and translation initiation-associated proteins, namely eIF1A, eIF3, eIF4G1, eIF6, and eEF1B, were downregulated. Hence, the initiation of the translation pathway was inhibited (Fig. 6). The results of LC–MS/MS also indicated that the expression of ABC transporter proteins ABCA8, ABCB1, and ABCG2 were downregulated, corresponding to our previous finding that sulbactam inhibits ABC transporters of *A. baumannii* and thus kills the bacterium [38]. Most ABC transporter families are transmembrane proteins, which are difficult to isolate and identify through total protein LC–MS/MS; hence, we used real-time RT-PCR to determine the effects of sulbactam on the mRNA expression of the ABC transporter proteins. The expression of ABC transporter proteins in breast cancer cells is highly heterogeneous [33, 44]; thus, we selected the ABCB superfamily, the ABCC superfamily, and ABCG2, which are strongly associated with drug resistance in breast cancer cells [23, 26, 32]. Based on the results of other studies and our PCR analysis, we selected ABCB1, ABCB2, ABCB8, ABCB10, ABCC1, ABCC2, ABCC3, ABCC4, ABCC5, ABCC10, and ABCG2, which exhibit high mRNA expression levels for precise real-time RT-PCR analysis.

Although the effects of sulbactam on these ABC transporters were different in MDA-MB-453 and MDA-MB-468 cells, we conclude that in the presence of sulbactam and doxorubicin, the mRNAs levels of the indicated ABC transporter proteins were evidently downregulated. ABCB1, ABCB5, ABCB8, ABCC1, ABCC2, ABCC3, and ABCG2 [22, 45–48] were considered to confer resistance to doxorubicin on the breast cancer cells. We further found that ABCB10, ABCC4, and ABCC5 in the MDA-MB-453 cells and ABCB10, ABCC5, and ABCC10 in the MDA-MB-468 cells also responded to sulbactam treatment. Studies have reported that ABCB5, ABCB8, ABCB10, ABCC2–5, and ABCC10 are overexpressed in breast cancer cells or are associated with breast cancer progression [44, 49–53]. Our doxorubicin efflux assay also indicated that in the presence of sulbactam, the retention time of doxorubicin in MDA-MB-453 and MDA-MB-468 cells was prolonged significantly. We used the computer simulation and found that sulbactam may compete with ATP for the ATP-docking sites of ABCB1, ABCB10, ABCC1, and MsbA, which exhibit structures similar to the ABCG2 (data not shown). This result provides a possibility how sulbactam inhibits the expression and function of ABC transporters, and this possibility is worthy to do more experiments to confirm it.

## Conclusion

In conclusion, this is the first study that using sulbactam in the mammalian cell. The combination of sulbactam and doxorubicin can enhance the cytotoxicity of doxorubicin in the breast cancer cells by inhibiting the transcription and initiation of translation associated proteins and ABC transporters, reducing their expression, and blocking the efflux of doxorubicin, thus triggering apoptosis in the breast cancer cells. From these results, sulbactam can be used in breast cancer treatment which can decrease the prescribed dose of doxorubicin to avoid the adverse effects.

## Abbreviations

MDR: multidrug resistance
ATP: adenosine triphosphate
ABC: ATP-binding cassette
MTT: 3-(4,5-dimethylthiazol-2-yl)-2,5-diphenyltetrazolium bromide
HER2: human epidermal growth factor receptor 2
TNBC: triple-negative breast cancer
P-gp: P-glycoprotein
PBP: penicillin-binding protein
DMEM: Dulbecco’s modified Eagle’s medium
FBS: fetal bovine serum
RPMI: Roswell Park Memorial Institute
IC_50_: the half maximal inhibitory concentration
RT-PCR: reverse transcription-polymerase chain reaction
PCR: polymerase chain reaction
PBS: phosphate buffered saline
SDS-PAGE: sodium dodecyl sulfate–polyacrylamide gel electrophoresis
ACN: acetonitrile
LC: liquid chromatography
MS/MS: tandem mass spectrometry
ICR: ion cyclotron resonance
ΔG: Gibbs free energy
ER: estrogen receptor
PR: progesterone receptor
Sul: sulbactam
Dox: doxorubicin
Vera: verapamil
